# Acute Effects of Milk vs. Carbohydrate on Bone Turnover Biomarkers Following Loading Exercise in Young Adult Females

**DOI:** 10.3389/fnut.2022.840973

**Published:** 2022-04-29

**Authors:** Joel L. Prowting, Lauren E. Skelly, Nigel Kurgan, Emily C. Fraschetti, Panagiota Klentrou, Andrea R. Josse

**Affiliations:** ^1^School of Kinesiology and Health Science, Faculty of Health, York University, Toronto, ON, Canada; ^2^Department of Kinesiology, Faculty of Applied Health Sciences, Brock University, St. Catharines, ON, Canada; ^3^Faculty of Applied Health Sciences, Centre for Bone and Muscle Health, Brock University, St. Catharines, ON, Canada

**Keywords:** dairy, milk, carbohydrate, female, acute exercise, bone biomarkers, plyometric exercise, resistance exercise

## Abstract

Dairy products and impact exercise have previously been identified to be independently beneficial for bone mineral properties, however, it is unknown how the combination of these two osteogenic interventions may alter acute bone turnover. Using a randomized crossover design, we compared the acute effects of consuming milk vs. an isoenergetic carbohydrate control beverage on bone biomarkers following loading exercise. Thirteen healthy female participants (Age = 20.3 ± 2.3y; BMI = 21.0 ± 1.1 kg/m^2^) consumed either 550 mL of 0% skim white milk (MILK) or 52.7 g of maltodextrin in 550 mL of water (CHO), both 5 min and 1 h following completion of a combined plyometric (198 impacts) and resistance exercise (3–4 sets/exercise, 8–12 reps/set, ∼75% 1-RM) bout. Venous blood samples were obtained pre-exercise, and 15 min, 75 min, 24 h and 48 h post-exercise to assess serum concentrations of bone resorption biomarkers, specifically carboxyl-terminal crosslinking telopeptide of type I collagen (CTX), receptor activator nuclear factor kappa-β ligand (RANKL), and sclerostin (SOST), as well as bone formation biomarkers, specifically osteoprotegerin (OPG) and osteocalcin (OC). When absolute biomarker concentrations were examined, there were no interaction or group effects for any biomarker, however, there were main time effects (*p* < 0.05) for RANKL, SOST, and OC, which were lower, and the OPG: OPG/RANKL ratio, which was higher at 75 min post-exercise compared with baseline in both conditions. In addition to assessing absolute biomarker concentrations at specific timepoints, we also evaluated the relative (% change) cumulative post-exercise response (75 min to 48 h) using an area under the curve (AUC) analysis. This analysis showed that the relative post-exercise CTX response was significantly lower in the MILK compared to the CHO condition (*p* = 0.03), with no differences observed in the other biomarkers. These results show that while milk does not appear to alter absolute concentrations of bone biomarkers compared to CHO, it may attenuate relative post-exercise bone resorption (i.e., blunt the usual catabolic response to exercise).

## Introduction

Osteoporosis is a disease that usually manifests in older adulthood and is characterized by a state of bone fragility and increased fracture risk due to losses in bone mineral density (BMD) and bone strength (1). Peak bone mass (PBM), achieved in early adulthood, is a strong predictor of osteoporosis later in life (2). Early implementation of strategies to increase PBM, particularly during the first 3 decades of life, when bone accrual is the greatest (3), may be one of the most effective preventative strategies for reducing the burden of osteoporosis as we age (2). Several low-cost, non-invasive osteogenic strategies have been identified, including engagement in loading exercise and nutritional modulation.

Regular exercise, particularly exercise that involves skeletal loading (4), is well known to be osteogenic (5). Plyometric and resistance exercise have both been shown to be particularly effective for both increasing PBM in adolescent/young adults and preventing bone loss in the elderly (4). The mechanical and metabolic stress of exercise stimulates the osteocytes embedded throughout the bone matrix to initiate the bone remodeling cycle in which osteoclasts first resorb the damaged bone tissue followed by the osteoblasts depositing new bone collagen (6). These processes are tightly coupled and regulated by several proteins and signaling molecules that may also be modulated by exercise and nutrition (6). For example, receptor activator nuclear factor kappa-β ligand (RANKL) and osteoprotegerin (OPG) oppose each other’s function, acting to increase and decrease osteoclast differentiation and function, respectively (7). Other proteins that directly affect bone remodeling include sclerostin (SOST), which is primarily secreted by osteocytes and acts on the Wnt pathway to impair bone formation by inhibiting osteoblast differentiation (8), and osteocalcin (OC) which is secreted by osteoblasts to aid in bone mineralization (9). The acute post-exercise time-course of changes in bone biomarkers is generally consistent with the bone remodeling process, whereby exercise typically elicits an initial transient increase in bone resorption [demonstrated by an increase in carboxyl-terminal crosslinking telopeptide of type I collagen (CTX)] (10–12), whereas bone formation markers are largely unresponsive initially, but can be upregulated later on (13). Although these are the typical responses observed, it is important to note that differences in exercise modality, intensity, duration, and participant training status may cause variations in acute biomarker responses (13). Since bone biopsies cannot be readily obtained in humans, measuring circulating biomarker concentrations to assess both the dynamic cellular processes that govern/modulate different stages of the bone remodeling cycle, as well as the end-products of such processes (e.g., CTX) can be insightful for determining acute responses to exercise and nutrition (7).

Maintaining an adequate dietary intake of bone-supporting nutrients is a strategy that has also been shown to support bone health (14). Dairy products provide many of these nutrients, and they contain more protein, calcium, magnesium, potassium, zinc, and phosphorus per calorie than any other food (15). It has been demonstrated through both observational and interventional research that consuming the recommended amount of dairy products can support bone health throughout the lifespan (16). For example, higher dairy intake was associated with elevated bone accrual during childhood (17) and adolescence (18), as well as with greater BMD in older individuals (19–21). In the context of exercise, previous research has indicated that dairy consumption alongside exercise training can push bone turnover status toward formation in young adult males (22), improve BMD and decrease bone resorption markers in female athletes (23), and induce favorable bone biomarker changes in pre-menopausal normal weight and overweight females (24–26). Bone is also acutely responsive to nutrition, as evidenced by changes in biomarkers following the manipulation of energy and carbohydrate intake (27, 28). However, despite positive findings following exercise training with concomitantly increased dairy consumption, little is known about the effect of post-exercise dairy consumption on acute bone cell responses following a single bout of loading exercise. Therefore, the purpose of this study was to examine serum levels of bone biomarkers following a single bout of combined plyometric and resistance (i.e., bone-loading) exercise followed by milk vs. carbohydrate consumption in normal weight, young adult females. Our primary outcome was examination of the absolute bone biomarker concentrations, and our secondary outcome was examination of the relative bone biomarker responses to post-exercise nutrition. We hypothesized that, relative to carbohydrate, post-exercise milk consumption would favorably influence bone turnover biomarkers (i.e., reduced resorption, enhanced/unchanged formation).

## Materials and Methods

This study was approved by both the Brock and York University Research Ethics Boards (#17-402 and #2019-045, respectively), met all human research standards of Canada’s Interagency Panel on Research Ethics and was registered at clinicaltrials.gov under the following identifier: NCT03615989.

### Participant Characteristics and Study Design

Thirteen young, healthy female adults were recruited from the Brock and York University (Ontario, Canada) student populations. Before informed consent was obtained, participants were initially screened to ensure they met the following inclusion criteria: (1) free of any medical conditions, (2) BMI 18.5–24.9 kg/m^2^, (3) recreationally active and not participating in a resistance training program, (4) no known allergy to dairy protein or lactose intolerance.

This study utilized a within-subject crossover design where, in a randomized order, participants completed 2 trials consisting of either: (1) exercise bout + carbohydrate (CHO) or (2) exercise bout + milk (MILK). All participants completed an initial lab visit consisting of baseline measurements and an exercise familiarization session before beginning their first trial. Prior to each trial, participants were instructed to arrive at the lab following an overnight fast and to refrain from alcohol and exercise for 48 h before and after the trial. If participants were not on hormonal contraceptives (*n* = 8), the trials were separated by a minimum of 4-weeks to allow for both trials to occur during the early follicular phase of the menstrual cycle. If participants were on monophasic hormonal contraceptives (*n* = 5), trials were separated by a minimum 2-week washout, and the trials occurred during hormone delivery.

### Baseline Testing and Familiarization Session

Upon study explanation and obtainment of written informed consent, baseline measurements were taken. These measures included height and weight, measured using a standard scale and stadiometer (with light clothing and shoes off), and body fat percent measured using bioelectrical impedance analysis (BIA; InBody 520 BIA system; Biospace Co., Inc. Los Angeles, CA, United States). To verify baseline physical activity and health status of the participants, the Godin Shephard leisure time physical activity questionnaire (29) and a general health and screening questionnaire were completed. In addition, a Physical Activity Readiness Questionnaire (2018 Par-Q +) (30) was administered to ensure each participant was safe to engage in physical activity. Participants were instructed to keep their diet consistent between trial sessions and to confirm this, were asked to record all their food and drink intake over a 2-day period for each trial (beginning on the day of the exercise bout). Dietary intakes were analyzed using the ESHA Food Processor Program (Food Processor SQL, ESHA Research, Salem, OR).

Participants then completed a familiarization session to learn how to correctly perform the trial exercises and to determine estimated 1 repetition maximum (1 RM) on three machine exercises (Chest Press, Seated Row, Leg Press), thus allowing determination of appropriate loads for the trial exercise bouts. Following a warm up, load was progressively increased in accordance with the American College of Sports Medicine exercise testing recommendations (31) until 5 repetitions or less could no longer be completed. All tests were completed by the same certified personal trainer. Estimated 1 RM was then calculated using the O’Connor calculation {1 RM = weight × [1 + (0.025 × # of reps)]} from the set with the lowest number of completed reps, a method that has been previously validated in females (32).

### Exercise Protocol

All exercise sessions began at approximately 9:00 a.m., were completed in a fasted state, and facilitated by a certified personal trainer. Participants completed the same exercise bout consisting of combined plyometric and resistance exercises for both trials, with each bout typically lasting approximately 70 min. The participants were asked to complete plyometric exercises, including broad jumps, pogo jumps, box drops, and explosive lunges (198 impacts/session), and resistance exercises, including chest press, leg press and seated row (3–4 sets/exercise, 8–12 reps/set, ∼75% 1 RM).

### Supplement Protocol

It was randomly determined (using a random number generator) which of the two isoenergetic and isovolumetric nutrition conditions the participants would complete first, with each being ingested 5–10 min following exercise cessation and again 1 h post consumption of the first trial drink. For the CHO condition, participants consumed 52.7 g of maltodextrin (∼200 kcals) mixed with approximately 550 mL of water plus a calorie-free, fruit flavored sweetener to enhance palatability. For the MILK condition, participants consumed 550 mL of 0% skim milk (Natrel fine filtered skim milk, Agropur, QC, Canada; 200 kcals, 20 g protein, 29 g carbohydrate).

### Blood Sample Collection Protocol

**FIGURE 1:**
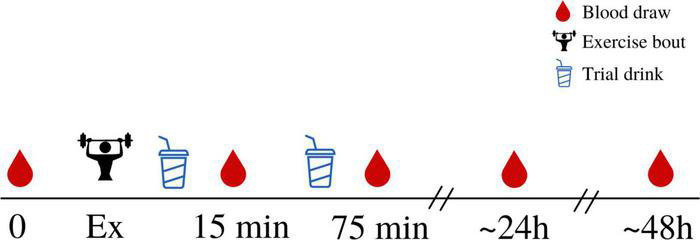
An overview of the experimental trial. Participants arrived for a baseline, rested, fasted blood draw before completing the exercise protocol. Immediately following completion of the exercise protocol, participants consumed the first trial drink (either MILK or CHO) which was then immediately followed by a blood draw (approximately 15 min after cessation of exercise). This was repeated 1 h later, with a second, identical, trial drink consumed 1 h after the first drink, and a second blood draw taken after the second drink had been consumed (approximately 75 min after cessation of exercise). Subsequent blood draws were taken 24 h and 48 h following exercise, in the fasted state.

Venous blood samples were collected from the median cubital vein by trained study personnel using a standardized venipuncture technique. Approximately 10 mL of blood was taken at each timepoint, which were pre-exercise (baseline), and 15 min, 75 min, 24 h, and 48 h post-exercise (Figure 1). After each draw, blood samples rested for 25 min (serum rested at room temperature, plasma rested at 4°C) before being centrifuged at 1,300 g and 4^°^C for 15 min. Serum and plasma were then aliquoted into small cryovial tubes and stored at –80^°^C until required for analysis.

### Bone Biomarker Assessment

Serum β–isomerized carboxy–terminal cross-linking telopeptides (CTX; cat# E-EL-H0960; Elabscience, China) and sclerostin (SOST; cat# DSST00; R&D, Minneapolis, MN) were analyzed in duplicate using enzyme linked immunosorbent assays (ELISA). The average intra-assay coefficients of variation for CTX and SOST were 11.8 and 5.7%, respectively, and the inter-assay coefficients of variation were 9.2 and 4.2%, respectively. Total osteocalcin (OC) and osteoprotegerin (OPG) were analyzed in duplicate using a multiplex human bone magnetic bead assay (cat# HBNMAG-51K; Millipore Corporation, Etobicoke, ON). The average intra-assay coefficients of variation were 8.5 and 6.3%, respectively, and the average inter-assay coefficients of variation were 2.9 and 3.9%, respectively. Serum RANKL was analyzed in duplicate using a single-plex RANKL magnetic bead assay (cat# HRNKLMAG-51K-01; Millipore Corporation, Etobicoke, ON). The average intra-assay coefficient of variation was 4.8% and the average inter-assay coefficient of variation was 4.6%.

### Statistical Analysis

Data were checked for normality using z-scores for skewness and kurtosis and non-normally distributed data were log transformed (RANKL). Individual missing values were dealt with by either using the same baseline concentration as their CHO trial if baseline values were missing (one value each for CTX, OC, OPG, RANKL in the same participant) or a “last observation carried forward” approach (one value for CTX; two values each for OC and OPG; three for RANKL; one value for SOST out of a total of 130 values per biomarker) (33). For the latter approach, 8 of 9 of these values were to replace missing 48 h values (carried forward from the respective 24 h value). In addition, one participant’s serum OC and OPG concentrations were below the assay detection limit at all timepoints and another participant’s serum RANKL concentrations were deemed invalid as they were all > 3SDs above the mean. Thus, the sample size was reduced for the analyses conducted on these variables (OC, OPG, RANKL *n* = 12; OPG/RANKL ratio *n* = 11). Average dietary intake data (Table 1) were missing for 2 participants, so the data from the 11 remaining participants were reported and analyzed between trial conditions using a paired samples *t*-test (with *p*-values considered significant at *p* < 0.05). Exercise volume load (sets × reps × weight) between the two conditions was also compared using a paired samples *t*-test (with *p*-values considered significant at *p* < 0.05).

**TABLE 1:** Average daily dietary intake during the MILK and CHO trials, based on analysis of 2-day food records (day of exercise and day post-exercise) including the provided trial drinks (*n* = 11).

Dietary variable	CHO	MILK	*p*-value
Energy (kcal)	1,958 (627)	1,873 (616)	0.46
Protein (g)	67 (27)	83 (31)	**0.01**
Carbohydrate (g)	274 (91)	243 (106)	0.07
Fat (g)	68 (30)	68 (24)	0.98
Vitamin D (IU)	47 (54)	408 (50)	**<0.001**
Calcium (mg)	561 (374)	1,073 (266)	**<0.001**
Iron (mg)	10 (4)	11 (6)	0.67
Magnesium (mg)	194 (145)	282 (113)	**0.01**
Potassium (mg)	1,768 (1,379)	2,754 (1,316)	**<0.001**

*Values are presented as mean (SD). Contribution of two CHO beverages: 401 kcals, 105 g carbohydrate. Contribution of two MILK beverages: 400 kcals, 40 g protein, 58 g carbohydrate, 720IU Vitamin D, 1,200 mg calcium, 1.44 mg iron, 160 mg magnesium, 1,680 mg potassium. Bold p-values denote statistically significant differences (p < 0.05).*

The absolute serum levels of all the bone turnover markers during CHO and MILK trials were compared using a two-way repeated measures analysis of variance (2RM-ANOVA), with condition (CHO and MILK) and time (baseline, 15 min, 75 min, 24 h, 48 h) both as within-participant factors. The Huynh-Feldt correction was used when data did not meet the assumption of sphericity. In the event of a significant main effect for time, *post hoc* testing was carried out involving pairwise comparisons using least significant difference (LSD) tests. Statistical analyses were performed using SPSS (Version 27.0 for Windows, Armonk, NY: IBM Corp.) with *p*-values considered significant at *p* < 0.05.

To evaluate our secondary objective, the net area under the curve (AUC) relative to pre-exercise was calculated for all biomarkers from the percent change data to assess short-term response following the post-exercise supplement (i.e., 75 min to 48 h) (34). The 15 min timepoint was excluded from this analysis because it was not affected by the supplement. The AUC for each biomarker was compared between trials using a paired-samples *t*-test for normally distributed data and a Wilcoxon’s matched-pairs signed rank test for SOST and OPG/RANKL ratio. The AUC calculations were performed using GraphPad Prism software 9.0 (GraphPad Software, La Jolla, CA, United States).

## Results

Thirteen participants completed both trials, and their baseline characteristics are shown in Table 2. Six participants completed the MILK trial before the CHO trial, while the remaining seven participants completed the CHO trial before the MILK trial.

**TABLE 2:** Participant characteristics measured at the start of the study (*n* = 13).

Participant characteristics	
Age (years)	20.3 (2.3)
Height (m)	1.6 (0.1)
Weight (kg)	56.6 (5.0)
BMI (kg/m^2^)	21.0 (1.1)
Body fat (%)	23.5 (3.3)

*Values are presented as mean (SD). BMI, body mass index.*

### Trial Exercise Volume Load

To ensure there were no differences in the exercise stimuli, we compared the exercise load between trial conditions. There was no significant difference in resistance exercise volume load (sets × reps × weight) performed during the exercise bout between trials (CHO trial: 5,487 ± 1,482 kg vs. MILK trial: 5,345 ± 1,485 kg; *p* = 0.25). In addition, for both the CHO and MILK trials, all participants completed 198 impacts/jumps per exercise session.

### Dietary Intake

To ensure nutrient intake during the trials was consistent, we examined 2-day food records with trial drinks included (*n* = 11) between trials, which are reported in Table 1. There were no significant differences between the habitual diets of the participants during the trials when analysis was performed with trial drinks removed (*p* > 0.05 for all variables), however, as expected, when the trial drinks were included, some differences in nutrients were noted (Table 1).

### Serum Levels of Bone Turnover Biomarkers

Absolute bone biomarker concentrations are shown in Table 3. There were no significant main effects for time or condition and no significant time-by-condition interactions for the absolute concentrations of CTX and OPG (*p* > 0.05). There was a significant main effect for time for OC (*p* = 0.01), SOST (*p* < 0.001), RANKL (*p* = 0.004) and the OPG/RANKL ratio (*p* = 0.02), but no significant main effects for condition or time-by-condition interactions (*p* > 0.05). *Post hoc* analysis of the main time effects indicated that compared to baseline and 15 min post-exercise, the concentrations of OC, SOST, and RANKL were significantly lower at 75 min post-exercise (*p* < 0.05). OC and RANKL returned to baseline levels at 24 h and 48 h post-exercise, however, SOST concentrations were significantly elevated at 24 h and 48 h post-exercise compared to both baseline and 75 min post-exercise (*p* < 0.05). In addition, RANKL concentrations were shown to be significantly below baseline at 15 min post-exercise (*p* < 0.05). The OPG/RANKL ratio was increased 15 min and 75 min post-exercise relative to baseline in both trials (*p* < 0.05), returning to baseline at 24 h.

**TABLE 3:** Absolute serum concentrations of bone biomarkers pre- and post-exercise during the carbohydrate (CHO) and milk (MILK) trials.

Analyte	CHO						Milk				RM-ANOVA		
	BL	15 min	75 min	24 h	48 h	BL	15 min	75 min	24 h	48 h	Cond	Time	Int
CTX (pg/ml)	221 (107)	212 (103)	246 (118)	238 (122)	208 (90)	254 (126)	250 (134)	224 (95)	234 (141)	240 (167)	0.61	0.92	0.08
OC (pg/ml)	16,807 (5,386)	17,041 (6,074)	15,557 (5,024) ***^ab^***	16,006 (5,946)	16,109 (5,131)	15,596 (5,228)	16,810 (5,811)	13,798 (4,781) ***^ab^***	16,897 (5,974)	14,589 (4,774)	0.20	**0.01**	0.31
SOST (pg/ml)	155 (80)	178 (84)	122 (59) ***^ab^***	176 (84) ***^ac^***	176 (66) ***^ac^***	164 (70)	181 (77)	140 (56) ***^ab^***	192 (87) ***^ac^***	193 (85) ***^ac^***	0.21	**<0.001**	0.85
OPG (pg/ml)	337 (133)	351 (145)	366 (150)	334 (75)	340 (78)	304 (68)	351 (80)	355 (65)	316 (85)	304 (72)	0.22	0.09	0.57
RANKL^†^ (pg/ml)	40 (21)	37 (31) ***^a^***	31 (35) ***^ab^***	41 (30) ***^c^***	41 (33) ***^c^***	47 (37)	39 (41) ***^a^***	34 (44) ***^ab^***	44 (38) ***^c^***	49 (38) ***^c^***	0.91	**0.004**	0.77
OPG/ RANKL ratio	11 (8)	15 (10) ***^a^***	22 (18) ***^a^***	11 (7) ***^bc^***	10 (5) ***^bc^***	8 (4)	13 (7) ***^a^***	20 (16) ***^a^***	10 (7) ***^bc^***	9 (7) ***^bc^***	0.20	**0.02**	0.89

*Values are mean (SD). CTX, SOST (n = 13); OC, OPG, RANKL (n = 12); OPG/RANKL ratio (n = 11). Post hoc analysis for combined trials (on the main time effects; bold letters): a, significant difference vs. pre-exercise; b, significant difference vs. 15 min post-exercise; c, significant difference vs. 75 min post-exercise using least significant difference tests (p < 0.05). ^†^Log-transformed data used for statistical analysis. BL, baseline; CTX, C-terminal crosslinked telopeptide of type-I collagen; OC, osteocalcin; SOST, sclerostin; OPG, osteoprotegerin; RANKL, receptor activator of nuclear factor-kβ ligand. Bold p-values denote statistically significant differences (p < 0.05).*

### Relative Post-exercise Bone Biomarker Response

**FIGURE 2:**
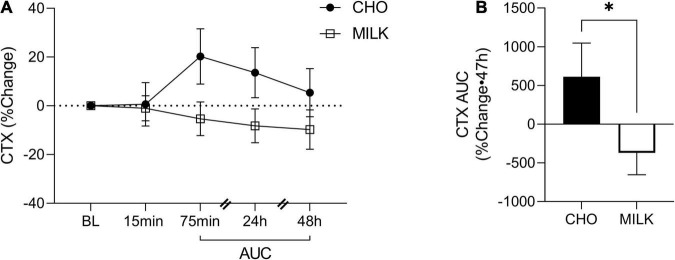
Percent change post-exercise **(A)** and area under the curve from 75-min to 48-h **(B)** for C-terminal crosslinked telopeptide of type-I collagen (CTX) in the CHO and MILK trials. Values are means ± SEM. *n* = 13. *Significant difference between conditions based on a paired *t*-test (*p* = 0.03). BL, baseline; AUC, area under the curve.

**FIGURE 3:**
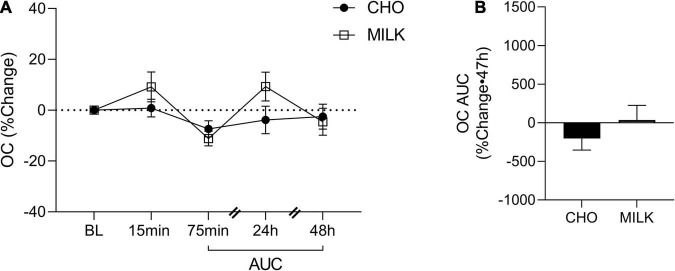
Percent change post-exercise **(A)** and area under the curve from 75-min to 48-h **(B)** for osteocalcin (OC) in the CHO and MILK trials. Values are means ± SEM. *N* = 12. BL, baseline; AUC, area under the curve.

**FIGURE 4:**
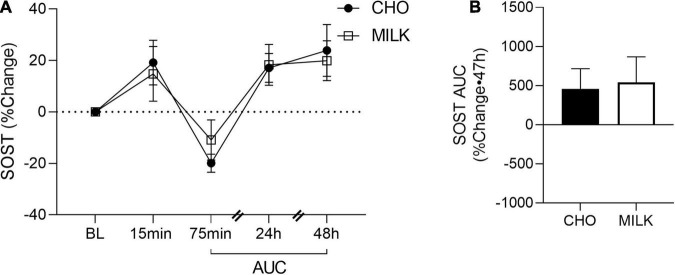
Percent change post-exercise **(A)** and area under the curve from 75-min to 48-h **(B)** for sclerostin (SOST) in the CHO and MILK trials. Values are means ± SEM. *n* = 13. BL, baseline; AUC, area under the curve.

**FIGURE 5:**
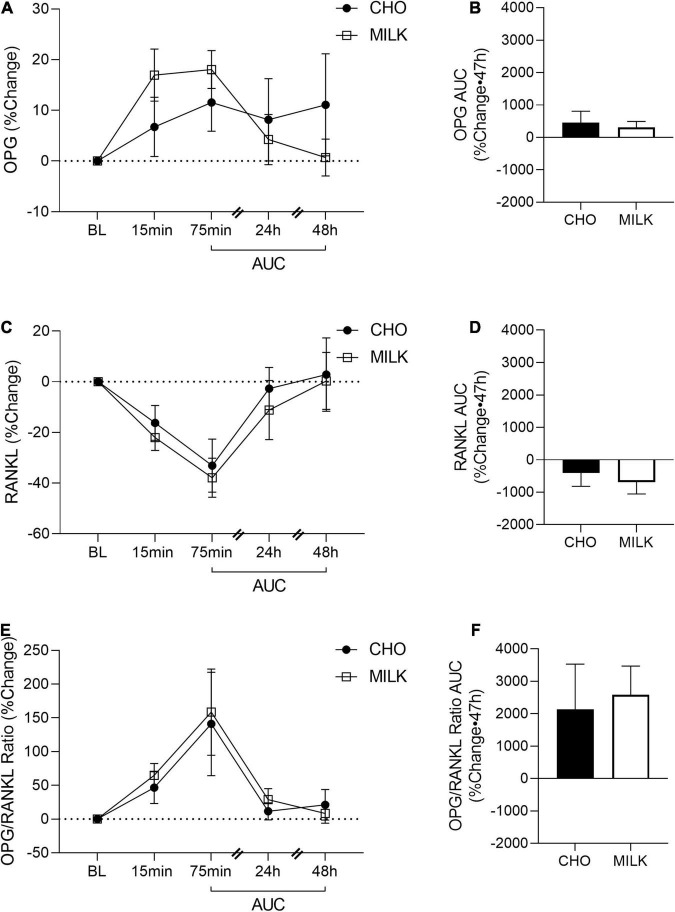
Post-exercise percent change in osteoprotegerin (OPG; **A**), receptor activator of nuclear factor-kB ligand (RANKL; **C**) and OPG/RANKL ratio **(E)** and area under the curve from 75-min to 48-h for OPG **(B)**, RANKL **(D)** and OPG/RANKL ratio **(F)** in the CHO and MILK trials. Values are means ± SEM. OPG and RANKL (*n* = 12); OPG/RANKL ratio (*n* = 11). BL, baseline; AUC, area under the curve.

To address the secondary objective of the study and examine the relative post-exercise and post-nutrition bone marker responses, we determined the net AUC relative to baseline from 75-min to 48 h post-exercise for each bone turnover marker using percent change values (35) (Figures 2A, 3A, 4A, 5A,C,E). The AUC analyses demonstrated that the CTX response to exercise was significantly lower in the MILK trial compared to the CHO trial (*p* = 0.03; Figure 2B). There was no significant difference in the post-exercise response between trials for OC (*p* = 0.39; Figure 3B), SOST (*p* = 0.54; Figure 4B), OPG (*p* = 0.72; Figure 5B), RANKL (*p* = 0.61; Figure 5D) or the OPG/RANKL ratio (*p* = 0.70; Figure 5F).

## Discussion

This is the first study to compare the influence of post-exercise milk vs. CHO consumption following plyometric and resistance loading exercise on acute bone biomarker responses in young adult females. We demonstrated that: (1) absolute biomarker concentrations were not differentially altered at any timepoint between the milk and CHO trials, (2) the relative post-exercise CTX response (indicative of bone resorption) was blunted following milk consumption compared to CHO consumption, and (3) a bout of resistance and plyometric loading exercise stimulated an acute bone cell response. Overall, these results suggest that milk (with its important constituent nutrients that support bone) may be a beneficial post-exercise nutritional choice and better than carbohydrates at reducing the typical acute elevations in bone tissue degradation that occur following exercise.

Acute pre-exercise dairy consumption has previously been shown by Haakonssen et al. (36) to attenuate bone resorption biomarkers following a single bout of endurance exercise in well-trained female cyclists. Utilizing a randomized crossover design, their trial consisted of a 90-min bout of intense cycling preceded by either a dairy containing meal (oats with calcium-fortified milk and yogurt) or a control meal (oats with water, tinned fruit, and nuts). The dairy condition displayed significantly reduced CTX concentrations immediately, 40 min and 190 min post-exercise, which is consistent with our findings showing that the relative CTX response was blunted by milk but not by CHO. Furthermore, neither our study nor theirs detected differences in the acute response of bone formation markers [OC, OPG, OPG/RANKL ratio in ours, and procollagen type 1 N-terminal Propeptide (P1NP) in theirs (36)] between the dairy and carbohydrate trials. Other studies have also demonstrated no acute effect of exercise on bone formation biomarkers (35, 37). We recently assessed the effect of Greek yogurt (3 servings/d) vs. carbohydrate consumption on bone biomarkers following 5-days of intense soccer training in adolescent female athletes (38). In this population, neither the intense exercise nor the Greek yogurt appeared to elicit any alterations in absolute bone biomarker concentrations over this period, although methodological differences, such as differences in exercise modality (5-d soccer training vs. single, high-load, high-impact exercise bout), limit the ability to directly compare findings. In addition, the adolescent elite female athletes previously assessed displayed much lower basal CTX concentrations compared to both the participants in the current study as well as to other young adult cohorts previously assessed (39, 40). This may have reduced the ability to detect any potential differences induced by Greek yogurt consumption on bone resorption because of a “floor effect.” Despite this, there were differences in the concentration of undercarboxylated OC (but not total OC) in the Greek yogurt condition compared to the carbohydrate condition, indicating that dairy may be uniquely affecting bone cell metabolism in the short-term. Research assessing the effects of acute dairy intake alone (i.e., not in conjunction with exercise) have also observed alterations in bone biomarker responses. For example, Hettiarachchi et al. (41) found that a calcium-fortified dairy supplement significantly reduced CTX and increased P1NP concentrations in the 4 h postprandial period, and Thomas et al. (42) observed significant reductions in CTX in the morning following consumption of 200 mL of skimmed milk fortified with extra calcium. We extend the literature in this area by analyzing the influence of dairy consumption on a variety of biomarkers associated with bone cell metabolism/turnover (specifically CTX, OC, SOST, OPG, and RANKL) and by assessing their responses following a resistance and plyometric exercise protocol. Although CTX was the only biomarker in which we observed significant differences in the relative response between trials, it is considered to be a robust biomarker for evaluating bone resorption [deemed to have higher specificity to bone metabolism and a smaller relative biological variability compared to other biomarkers (43)], and thus these nascent findings that suggest a benefit of post-exercise milk on bone turnover are promising. Collectively, although the data are limited, it currently appears that dairy consumption (either alone or in close proximity to an exercise bout) can acutely attenuate bone resorption in humans.

Despite the paucity of literature specifically evaluating the effect of dairy on post-exercise bone biomarker responses, there is evidence that nutritional status and/or acute feeding of other nutrients can influence bone biomarkers. Sale and colleagues (35) found that both CTX and P1NP responses were attenuated for up to 2 h post-exercise with CHO feeding compared to a water control (when assessed using a similar post-exercise AUC analysis to ours), and concluded that CHO consumption during exercise acutely blunts bone turnover in the hours (up to 2 h post) following an exercise bout. The same group, in two additional studies, observed that trained males consuming CHO before, during, and after high-intensity interval running (vs. reduced CHO + high fat, and reduced CHO/energy conditions) (44) and trained adult males consuming CHO + whey protein (a component of dairy protein) immediately post-exercise vs. a water control (45), displayed an attenuated CTX response for up to 3 h post-exercise. When comparing the effect of post-exercise whey protein vs. carbohydrate consumption on bone turnover biomarkers, whey protein has been shown to provide greater reductions in bone resorption (CTX) in athletic samples of swimmers (39) and mountain bikers (37). Furthermore, there are also data showing attenuated CTX with acute calcium supplementation vs. a water or saline control following prolonged strenuous endurance exercise (46, 47). In our study, dietary calcium intake was found to be significantly higher during the MILK vs. the CHO trials, so it is possible that differences in calcium consumption contributed to the blunted relative post-exercise CTX response in the MILK condition. It is worth noting, however, that the previous studies (46, 47) showing a benefit of calcium consumption on bone resorption have done so following an intense endurance exercise bout (1 h in duration) in which there were likely significantly greater calcium losses through sweat than what would have occurred with our exercise protocol. At this time, it remains unclear how isolated calcium supplementation compares to the energy containing, wholefood dairy product we supplied. Therefore, our observation that milk appeared to blunt CTX over the post-exercise period in comparison to an isoenergetic CHO condition and given the previous findings of CHO on the acute bone biomarker responses, it may be prudent to at least consume energy post-exercise, but it is likely more favorable to consume milk (that contains a combination of energy, protein, and bone-supporting nutrients like calcium) post-exercise compared to carbohydrate/energy alone. Although we did not investigate a non-energetic post-exercise control (e.g., water), future studies should consider including this type of trial, and/or a calcium + water trial to compare the relative contribution(s) of energy and other potential functional ingredients within dairy foods on the acute bone response to exercise.

Despite only seeing differences in the relative CTX response between the nutritional interventions, we did observe various time effects elicited by our exercise bout, indicating that a single bout of intense, high load resistance, and plyometric exercise effectively stimulated a response within bone tissue. We have previously demonstrated that plyometric exercise (without post-exercise nutrition) can induce acute bone biomarker changes (including CTX, P1NP, SOST, OC) in both adult and adolescent females (48, 49). Specifically, we have consistently shown an immediate (5 min) post-exercise increase in SOST in studies of both males (50) and females (40, 48, 49, 51). In the present study, SOST tended to increase at 15 min post-exercise (*p* = 0.11), but the smaller sample size and/or the later sampling time (i.e., 5 min vs. 15 min) may have contributed to the observed non-significant increase. Moreover, we found that SOST, OC, and RANKL concentrations were suppressed 75 min post-exercise, whereas the OPG/RANKL ratio was significantly increased at this timepoint. With acute exercise, we may expect to see an increase (13) or no difference from baseline (52) in bone resorption markers at ∼1 h post-exercise, with bone formation markers unchanged (although bone biomarker responses in previous acute exercise research have been inconsistent) (12, 13, 53). It is likely that our energy containing interventions (both MILK and CHO) influenced the biomarker responses at 75 min post-exercise, resulting in a general shift of bone metabolism away from resorption and toward bone formation. Furthermore, our bone biomarker response is unknown in the later acute post-exercise period (i.e., between 75 min and 24 h). Future research is required to further understand the interactions between loading exercise and energy/nutrition intake on bone biomarker responses, as well as to characterize their responses to loading exercise at different sampling timepoints (i.e., the intermediate response between 75 min and 24 h).

Dairy foods may be uniquely suited to enhance bone metabolism because they are a rich source bone-supporting nutrients, including energy, protein, calcium, and other micronutrients. Despite this, the mechanisms through which dairy products appear to alter bone cell metabolism are not well understood. This likely relates to our current inability to directly assess the cellular activity and pathways activated in human bone tissue following an acute stimulus such as exercise. Initial evidence suggests that bioactive peptides/components in dairy foods (e.g., milk-based protein, milk growth factors like IGF-1, lactoferrin, caseinophosphopeptides, glycomacropeptides) can act directly on osteoblastic (Wnt and Bone Morphogenic Protein/Transforming Growth Factor-β) and/or osteoclastic [by controlling production of RANKL and Macrophage-Colony Stimulating Factor (M-CSF)] signaling pathways (54). In this study, we did not observe any significant differences in the acute post-exercise response of circulating RANKL or OPG between nutrition conditions, so it remains unclear through which mechanism milk may have acted to blunt bone resorption. More research is warranted to uncover the mechanistic effects of these dairy-specific bioactives as well as to determine how different dairy products may influence bone metabolism (i.e., fermented dairy products like yogurt have greater concentrations of certain bioactive peptides, and thus may confer even more potent modulation of bone tissue mechanisms). Furthermore, as discussed in this new and relevant review by Dolan and colleagues (13), the relationship between acute exercise/nutrition related bone biomarker responses and long term positive changes in BMD and bone microarchitecture remains unclear. For example, while it is interesting that milk appeared to blunt the CTX response compared to CHO in our study [and that nutrition/energy provision appears to blunt acute bone resorption in other exercise studies (35, 38, 44, 45)], it may be valuable for future work to extend the observations over a longer duration within the same participants to help determine the link between acute/short-term biomarker responses and longer-term bone outcomes. Chronic studies involving combined exercise and dairy intake have previously demonstrated both improved structural (55) and biomarker (22, 56–58) outcomes. Indeed, Bridge et al. (22) observed a blunted bone resorption (CTX) response following just 1 week (3 bouts) of resistance + plyometric exercise (similar to the exercise bout in this study) with Greek yogurt consumption, which was followed, after 12 weeks, by a significant increase in bone formation (P1NP) vs. those consuming CHO post-exercise (although no BMD/structural outcomes were assessed).

Our study had several strengths, including the use of a crossover design to limit the influence of inter-individual variability, the assessment of multiple biomarkers involved with different aspects of the bone remodeling cycle, and the measurement of biomarkers at multiple timepoints over a 48 h period, allowing for AUC analyses and thus evaluation of the cumulative biomarker response over the entire post-exercise period. Also, we believe performing this research in a population of untrained young adult females is a strength of our study. Not only is this due to the general under-representation of female participants in nutrition and exercise research (59, 60), but also because osteoporosis preferentially affects females compared to males (61).

Our study also had several limitations. First, our sample size was relatively small, although we believe that the crossover design chosen helped to mitigate this concern. Secondly, the measurement and assessment of circulating biomarker concentrations to evaluate acute bone metabolism has its own inherent/established limitations (7, 62), however this currently remains the gold-standard option to assess acute bone responses to exercise and/or nutrition (13). Another potential limitation with systemic biomarker assessment is that there may be site-specific skeletal changes in turnover/metabolism following loading exercise that our systemic measures are not sensitive enough to differentiate. It is therefore possible that the biomarker changes that we are seeing underrepresent meaningful acute changes at specific skeletal sites.

## Conclusion

This study advances our understanding of how nutrition can modulate acute bone metabolism following exercise by comparing acute bone biomarker responses to exercise with milk or CHO consumption for the first time. In conclusion, despite no between trial differences in absolute bone biomarker concentrations, our study demonstrated that the consumption of milk following resistance and plyometric exercise, in untrained, healthy adult females, resulted in a greater relative acute suppression of bone resorption in comparison to an isocaloric carbohydrate drink. This novel finding suggests that milk may be a better post-exercise nutritional option (compared to CHO alone) for blunting the initial catabolic effect of exercise on bone. Future research is required to determine how dairy specifically modulates physiological responses within bone, including elucidating which component(s) of dairy are primarily responsible for these observed differences. In addition, RCTs are needed to examine long term effects of these interventions on bone.

## Data Availability Statement

The data sets presented in this article are not readily available. Requests to access the data sets should be directed to AJ, ajosse@yorku.ca.

## Ethics Statement

This study involving human participants was reviewed and approved by the Brock University Research Ethics Board—reb@brocku.ca and the York University Office of Research Ethics—re@yorku.ca. The participants provided their written informed consent to participate in this study.

## Author Contributions

AJ and PK: study design conceptualization. JP, LS, NK, EF, PK, and AJ: data analysis, interpretation, and drafting and revising the manuscript. All authors approved of the final version of this manuscript.

## Conflict of Interest

AJ reports consultant/speaker fees from Dairy Farmers of Canada. LS reports salary support from Dairy Management Inc. For projects outside the present work. AJ and PK report grant funding from Dairy Farmers of Canada, Dairy Management Inc., and non-financial support from Danone and Parmalat. LS reports grant funding from Dairy Farmers of Canada. The remaining authors declare that the research was conducted in the absence of any commercial or financial relationships that could be construed as a potential conflict of interest.

## Publisher’s Note

All claims expressed in this article are solely those of the authors and do not necessarily represent those of their affiliated organizations, or those of the publisher, the editors and the reviewers. Any product that may be evaluated in this article, or claim that may be made by its manufacturer, is not guaranteed or endorsed by the publisher.
